# Hospital Dinners and Annual Meetings

**Published:** 1906-05-26

**Authors:** 


### Hospital Dinners and Annual Meetings.

The most general form of raising a round sum for a hospital is to hold an anniversary dinner. These dinners usually follow one routine course, and it is a little remarkable that no attempt has been made to enliven the average dullness of their proceedings, or to attract to them the larger and more intelligent supporters of each institution, so as to quicken the interest in the management and secure an increasing number of workers genuinely interested in the hospital and the work which it is doing for the least fortunate of the race. Why, for instance, should the cinematograph not be introduced as a means of providing a very instructive and interesting entertainment, which might offer great educational advantages if it was made to bring out clearly the developments in the methods of treatment and the improvements in buildings and accommodation?

It is a good thing in our judgment that the more influential members of the Royal family should cause it to be understood that if one of their number is to preside at a festival dinner a supreme effort must be made to raise such a sum as will substantially aid the institution and make the occasion memorable in its history. We have no patience however with the class of guests who struggle to obtain an invitation to be present on such an occasion, but who fail to put in an appearance when, as happened last week, a Royal personage is prevented from attending at the last moment owing to domestic affliction. The task of the secretary in organising the attendance at festival dinners and making them financially successful is difficult indeed. But what are we to think of people who abstain from attendance, instead of making a point of being present in order to display their loyalty and sympathy in such circumstances?

Festival dinners should be held as rarely as possible, and a reform in the existing procedure should be instituted so as to make them generally popular with the best supporters of a voluntary hospital. At present the speeches are often too long and not infrequently far too numerous. We incline to the feeling that they should never exceed six, including the Royal toasts, and that it would be far better to arrange to adjourn to another room at the conclusion of the dinner, to let the chairman's speech on behalf of the hospital take the form of an explanatory statement, accompanied by cinematograph pictures of the hospital and its work, and to secure that the musical entertainment should be of the very best. In this way not only would the festival dinner become an important social function, but an opportunity might be afforded to the active members of the committee to get into close touch with some of the more important supporters and governors of the hospital.

It would be infinitely cheaper, and we incline to think, having regard to the modern habits of the people of these islands, infinitely more popular, for the chairman to hold an "At Home" at the hospital once a year on the occasion of the annual meeting, and to take that opportunity to bring in new money in the shape of subscriptions and donations to as large an extent as possible. Under such a system the festival dinner could be made a really important occasion, because of its infrequency, and the sum so raised, if the hospitals would co-operate so as to cut down the number of dinners to reasonable proportions each year, might constitute a quinquennial or triennial fund so large as to secure the liquidation of any accumulated deficiencies between current income and current expenditure.

We are well aware that the hospitals cannot altogether dispense with the festival dinner. When, however, during two or three of the busiest months of the year those who are genuinely interested in hospitals receive five or six invitations in one week, and where, as in one case, the number of these dinners on a given evening have exceeded ten, as one gentleman pointed out to us recently, the necessity for a change of plan in the interests of the voluntary hospitals is demonstrably proved. The guests who are in the greatest demand are usually the busiest of men, and it is a calamity to chill the ardour of zealous supporters by overdoing a method of raising money, which, at the best, should only be resorted to to meet an emergency, or to enable funds to be raised for some important development. The mere expenditure upon postage and printing, the output of energy required at the hands of the chairman and secretary, who have to put aside everything else in order to make these dinners a success, represent an actual value in hard cash which can only be justified by results which it is often impossible to obtain, owing to the multiplicity of dinners that now take place on behalf of charities each year. We are confident the more this matter is looked at and tested on its merits, the more must the wiser heads amongst our hospital managers be convinced, that the time has come for a reconsideration of the whole business, with a view to more economical administration and the attainment of larger and better financial results.

